# TiO_2_ Catalyzed Dihydroxyacetone (DHA) Conversion in Water: Evidence That This Model Reaction Probes Basicity in Addition to Acidity

**DOI:** 10.3390/molecules27238172

**Published:** 2022-11-24

**Authors:** Insaf Abdouli, Frederic Dappozze, Marion Eternot, Chantal Guillard, Nadine Essayem

**Affiliations:** Institut de Recherches sur la Catalyse et l’Environnement de Lyon, UMR 5256, CNRS, Université Claude Bernard Lyon 1, IRCELYON, F-69626 Villeurbanne, France

**Keywords:** titanium dioxide, dihydroxyacetone, CO_2_ and NH_3_ microcalorimetry, FTIR of pyridine adsorption, acidity, basicity

## Abstract

In this paper, evidence is provided that the model reaction of aqueous dihydroxyacetone (DHA) conversion is as sensitive to the TiO_2_ catalysts’ basicity as to their acidity. Two parallel pathways transformed DHA: while the pathway catalyzed by Lewis acid sites gave pyruvaldehyde (PA) and lactic acid (LA), the base-catalyzed route afforded fructose. This is demonstrated on a series of six commercial TiO_2_ samples and further confirmed by using two reference catalysts: niobic acid (NbOH), an acid catalyst, and a hydrotalcite (MgAlO), a basic catalyst. The original acid-base properties of the six commercial TiO_2_ with variable structure and texture were investigated first by conventional methods in gas phase (FTIR or microcalorimetry of pyridine, NH_3_ and CO_2_ adsorption). A linear relationship between the initial rates of DHA condensation into hexoses and the total basic sites densities is highlighted accounting for the water tolerance of the TiO_2_ basic sites whatever their strength. Rutile TiO_2_ samples were the most basic ones. Besides, only the strongest TiO_2_ Lewis acid sites were shown to be water tolerant and efficient for PA and LA formation.

## 1. Introduction

The replacement of petroleum products by biomass-derived ones has been attracting growing attention in recent years. In this regard, the valorization of biomass and its derivatives into value-added products has been largely studied by means of different processes, especially hydrothermal ones. For these processes, homogeneous and heterogeneous acid-base catalysts were used to catalyze specific reactions steps such as hydrolysis, retro-aldolization, dehydration, etc. However, in hydrothermal conditions, solid acids and bases may be inhibited by water.

Different physicochemical methods are now well established to characterize the original acid-base properties of heterogeneous catalysts. Most of them are performed in the gas phase such as temperature-programmed desorption (TPD) of probe molecules [1,2,3,4]; adsorption of probe molecules monitored by microcalorimetry [1,3,4,5] or infrared spectroscopy (FTIR) [3,4,6], etc. Nevertheless, these techniques characterize heterogeneous catalysts in gas phase, conditions completely different from the hydrothermal medium where acid or basic sites are very often inhibited by water [7,8].

Besides, the use of model reactions is an effective method since they are conducted under conditions as similar as possible to the reactional ones. Model reactions, whose mechanisms are well known, were largely applied in the gas phase such as light alkanes or alkenes isomerization or alcohol dehydration or dehydrogenation [9,10,11,12]. Each pathway is specifically catalyzed by acid and/or basic sites. The reactions rates, the products distribution and the mechanism involved may give insight on the nature of the active sites and/or on their density or strength. In the liquid phase, the use of model reactions is less common, but it is increasing for the characterization of acid/base properties, in particular in the presence of water [13,14,15,16,17,18]. For instance, cyclohexanol dehydration was previously applied to investigate the efficiency of various solid acids [17,18], and acetone aldolization in the presence of increasing water amounts was applied to characterize the water tolerance of hydrotalcites’ basic sites [8].

**Scheme 1:**
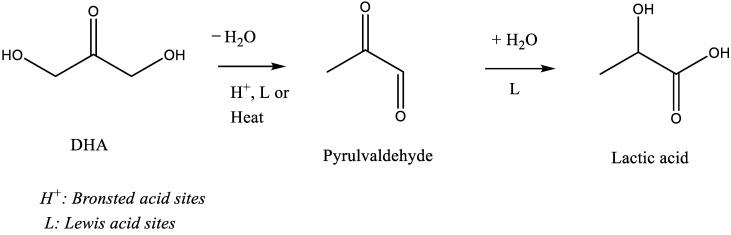
Widely accepted route for DHA conversion into lactic acid via pyruvaldehyde formation.

Recently, the production of lactic acid (LA) from dihydroxyacetone (DHA) or its derivative, pyrulvaldehyde (PA), has been extensively investigated to evaluate the Lewis acid sites water tolerance of various solid acid catalysts and was also seen to be efficient to determine their acid sites types (Lewis vs. Brønsted) [16,19,20,21,22,23,24]. It is generally accepted that the reaction proceeds in two steps. The first step, where the DHA undergoes dehydration to produce PA, is either an acid catalyzed step (Brønsted and/or Lewis) at low temperature < 100 °C or thermally induced at higher temperature [16,23]. The second step that converts PA into LA is known to be catalyzed by Lewis acid sites only (Scheme 1) [16,19,21,23].

Many homogenous [16] or heterogeneous catalysts such as Nb_2_O_5_ [20,22,24], zeolites [18,19,25,26], phosphates [23], ZrO_2_ [21] or doped silica [27] have been studied. Several earlier studies investigated the acid properties of TiO_2_ samples using this model reaction [22,24,28] and one can note some disagreement. Anatase TiO_2_, prepared by sol–gel synthesis, was shown to be a very active solid acid to produce lactic acid from pyruvaldehyde due to its high amount of Lewis acid sites, which, unlike Nb_2_O_5_, would not be inhibited by water adsorption [24]. Besides, Nb_2_O_5_ was described elsewhere to be more efficient than a commercial anatase/rutile TiO_2_ [22].

It can also be noted that, despite the very broad applications of TiO_2_, especially as support and in photocatalysis, there is no established agreement on the original acid base properties of a reference TiO_2_ material such as TiO_2_ P25 in term of nature, number of sites and strength, investigated with the usual technics in gas phase. For instance, Brønsted acid sites were not detected by FTIR of pyridine adsorption over P25 [29] or on pure anatase TiO_2_ prepared by sol–gel synthesis [24,28,30,31] whereas few others mentioned the presence of Brønsted acidity over P25 or other commercial TiO_2_ anatase by NH_3_ adsorption monitored by FTIR [32,33]. On the other hand, the basicity of TiO_2_ has been scarcely studied. One can mention the earlier ones of Watanabe et al. that revealed its bifunctionality, the presence of acid and basic sites over an anatase TiO_2_, by gas phase TPD experiments [34].

More generally, there is a lack of literature on the original acid-base properties of TiO_2_ as a function of its crystalline structure and/or morphology for exploring possible correlations with their (photo)catalytic activities. Therefore, probing the acid-base properties of TiO_2_ samples with different crystalline structures and/or morphologies, in water, to highlight correlations with their photocatalytic behaviour, would represent a real breakthrough.

In this work, with the objective of understanding the catalytic behaviour of different TiO_2_’s crystalline structure (anatase/rutile) with variable specific surface areas, we studied their catalytic performances in the model reaction of DHA conversion in water. In parallel, we measured the original acidity and basicity of six selected commercial TiO_2_ samples using well-established gas phase methods: namely, pyridine, NH_3_ and CO_2_ adsorption monitored by FTIR and microcalorimetry to get reliable data on the nature, density and strength of the original acidity and basicity of each TiO_2_ samples in the absence of moisture.

In this work, we use the model reaction of DHA conversion in the aqueous phase to probe the catalyst’s Bronsted/Lewis acidity, while demonstrating that this reaction can also probe the basic properties leading to DHA condensation into hexoses. This conclusion was supported by the studies of different commercial TiO_2_ samples with variable acid-base properties as established by the usual gas phase methods and by the study of reference acid and base catalysts, i.e., NbOH and MgAlO

## 2. Results and Discussion

### 2.1. Catalysts Characterization

**Figure 1:**
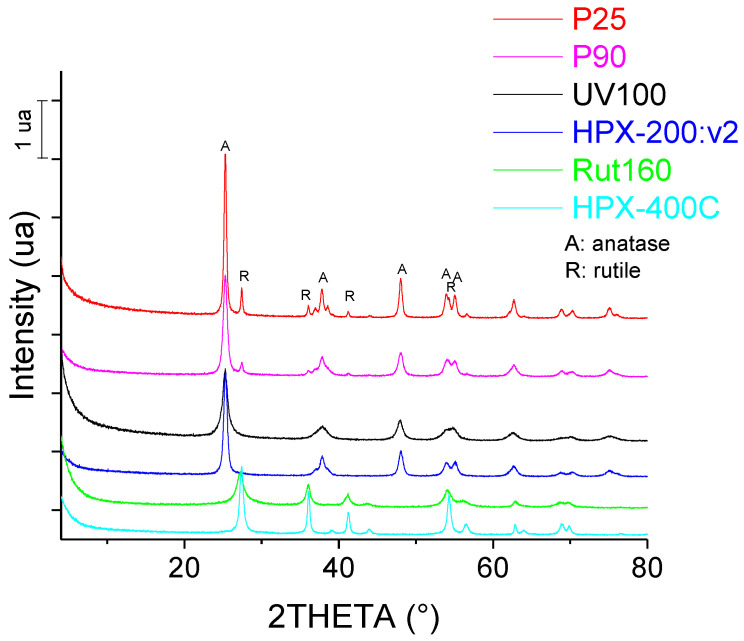
Diffractogram of commercial TiO_2_ samples.

The XRD patterns of the six commercial TiO_2_ catalysts are shown in Figure 1. It is confirmed that P25 and P90 are mixtures of anatase and rutile crystalline phases, UV100 and HPX-200/v2 are pure anatase and Rut160 and HPX-400C are pure rutile. From XRD measurements, the average size of TiO_2_ crystallites was found to be around 8 nm for UV100 and Rut160, ~12 nm for P90 and HPX-200/v2, 16 nm for HPX-400C and around 20 nm for P25 (Table 1). The crystallite size of UV100 and P25 were in agreement with those determined by Arana et al. [32].

The BET surface areas are roughly related to the size of the TiO_2_ crystallites, which indicates that they are mainly developed by the external surfaces of the particles.

**Figure 2:**
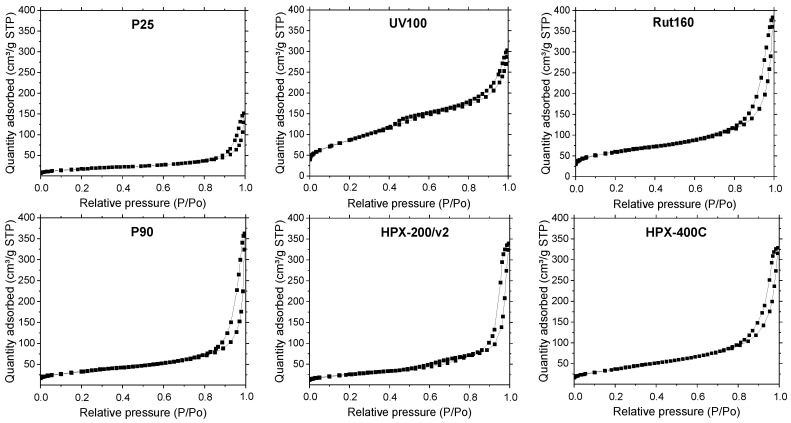
N_2_ adsorption/desorption isotherm of the commercial TiO_2_ samples. Catalyst pretreatment: 300 °C for 2 h under vacuum (heating rate: 5 °C.mn^−^^1^).

The isotherms shown in Figure 2 present the same feature, the presence of a hysteresis loop at high relative pressure, linked with the presence of large mesopores, above 3–5 nm. UV100 and Rut160 present also micropores as indicated by the significant N_2_ adsorption at very low relative pressure. As shown in Table 1, the highest BET surface areas were observed for UV100 (300 m^2^.g^−1^) and Rut160 (175 m^2^.g^−1^), probably due to their smallest crystallites and the presence of micropores. The P25 showed the lowest surface area (55 m^2^.g^−1^), whereas the rest of the samples, i.e., P90 (mixture of anatase and rutile), HPX-200/v2 (anatase) and HPX-400C (rutile), exhibited almost similar surface areas close to 100 m^2^.g^−1^.

### 2.2. Catalysts’ Acidity

**Figure 3:**
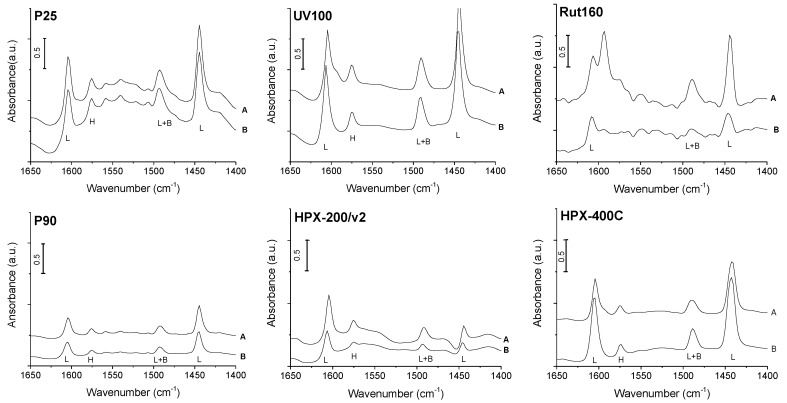
Infrared spectra of pyridine adsorption on P25, P90, UV100, HPX-200/v2, Rut160 and HPX-400C: (A) spectra of catalysts saturated with pyridine vapor at ambient temperature, then desorption of pyridine for 1 h at ambient temperature; (B) spectra after pyridine desorption at 150 °C for 1 h (L: characteristic vibration of Lewis acid sites, B: characteristic vibration of Brønsted acid sites, L + B: vibration common to Lewis and Brønsted acid sites, H: vibration of pyridine linked by hydrogen bonds). Conditions: Prior to pyridine adsorption, TiO_2_ self-supported pellets were vacuum treated for 1 h at 150 °C (reference spectra), normalized spectra to 100 mg.

The catalysts’ acidity was first studied by pyridine adsorption followed by FTIR to identify the nature of the acid sites, Lewis vs. Brønsted. The different FTIR spectra obtained after pyridine adsorption on TiO_2_ dehydrated at 150 °C, then vacuum treated at ambient temperature and 150 °C, are shown in Figure 3. All the catalysts exhibited bands at 1445 and 1610 cm^−1^ that correspond to the vibrations characteristic of pyridine coordinated to the catalysts’ Lewis acid sites. The spectra also show a band at 1490 cm^−1^ common to a vibration mode of pyridine coordinated to the catalysts’ Lewis acid sites and to pyridinium ions. The band observed at 1575 cm^−1^ was previously ascribed to a vibration of pyridine linked by hydrogen bonds to the catalysts’ surface [35]. However, no significant peak at 1545 cm^−1^ nor at 1640 cm^−1^, corresponding to the vibrations characteristic of the pyridinium ions, formed in the presence of Brønsted acid sites, are clearly observed. Over Rut160 and P25, if present, this vibration is hardly detected from the baseline. Thus, from our FTIR study of pyridine adsorption on the TiO_2_ samples dehydrated at 150 °C, one can conclude that all the six TiO_2_ samples have only Lewis acid sites in agreement with previous investigations [24,29]. Our study does not demonstrate the presence of Brønsted acidity on these anatase and/or rutile TiO_2_ samples, in contrast to others [32].

The study of the catalysts’ acidity was then completed by NH_3_ adsorption monitored by microcalorimetry coupled to the NH_3_ isotherm measurements: a precise and reliable technique to determine the total number of acid sites and their acid strength distribution. As determined by the adsorption of pyridine followed by FTIR, all the catalysts have only Lewis acid sites; thus, the adsorption of NH_3_ monitored by microcalorimetry measures the amount and the density of the Lewis acid sites.

**Figure 4:**
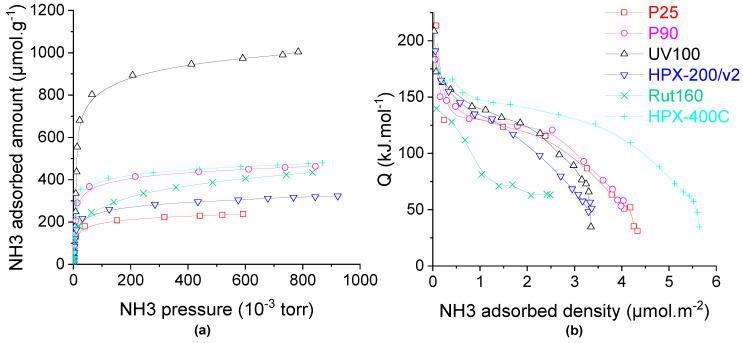
Ammonia adsorption on the TiO_2_ samples: (**a**) isotherms and (**b**) calorimetric curves. Conditions: TiO_2_ samples pre-treated at 150 °C under secondary vacuum for 5 h, NH_3_ adsorption performed at 80 °C.

The isotherms of NH_3_ adsorption on all the samples dehydrated at 150 °C are compared in Figure 4a and the corresponding calorimetric curves are displayed in Figure 4b. The pure anatase TiO_2_, UV100, which also exhibits the highest BET surface area of 300 m^2^.g^−1^, has more than twice the acid sites (~900 µmol.g^−1^) in comparison to other materials. On the other hand, the P25, which is made of a mixture of anatase and rutile phases with the lowest BET surface area, has the lowest amount of the acid sites, ~200 µmol.g^−1^. Total acid sites amounting to between 240 and 410 µmol.g^−1^ are measured on all other TiO_2_ samples which also present intermediate BET surface areas. Since the samples have quite different specific surface areas, the calorimetric curves are compared as a function of the acid site density, expressed in µmol NH_3_ per square meter (Figure 4b). The calorimetric curves of all the catalysts except Rut160 and HPX-400C show quite an equivalent pseudo plateau which indicated the presence of many acid sites of homogeneous acid strength with heat of NH_3_ adsorption between 120 kJ.mol^−1^ and 140 kJ.mol^−1^ for P25, P90, HPX-200/v2 and UV100. HPX-400C presents a calorimetric curve well above the previous ones, with the presence of stronger acid sites, of homogeneous strength, characterized by a longer pseudo-plateau between 155 and 125 kJ.mol^−1^. In contrast, the calorimetric curve of Rut160 shows a continuous decrease of the heat of ammonia adsorption with the ammonia coverage which indicates the presence of a more heterogenous surface in terms of strength with significant lower acid strength.

Except Rut160, all the TiO_2_ samples present few strong acid sites with heat of ammonia adsorption higher than 150 kJ.mol^−1^. Thus, to tentatively understand the effect of catalysts’ crystalline phase and/or the crystallites’ size on the TiO_2_ acidity, the acid sites densities (acid sites amount divided by the catalyst’s surface area) are compared in Table 2. As shown in Table 2, catalysts containing only anatase (UV100 and HPX-200/v2) or mainly anatase phase (80% for P25 and 92% for P90) have acid sites densities around 3 µmol.m^−2^ with close acid strength distribution. This would indicate that the Lewis acidity of anatase TiO_2_ would not be sensitive to the structure (crystallite size). On the contrary, one can observe quite different acid site density on pure rutile TiO_2_: around 1.4 µmol.m^−2^ and 5 µmol.m^−2^ for Rut160 and HPX-400C, respectively. Since HPX-400C with crystallites’ size around 16 nm is more acidic than Rut160 with smaller particles (8.5 nm), one could suggest that the acidity of rutile TiO_2_ would be structure sensitive and the Ti^4+^ exposed on the faces of the larger crystallites of the rutile TiO_2_ HPX-400C would make it more acidic than the rutile TiO_2_ Rut160.

### 2.3. Catalysts Basicity

**Figure 5:**
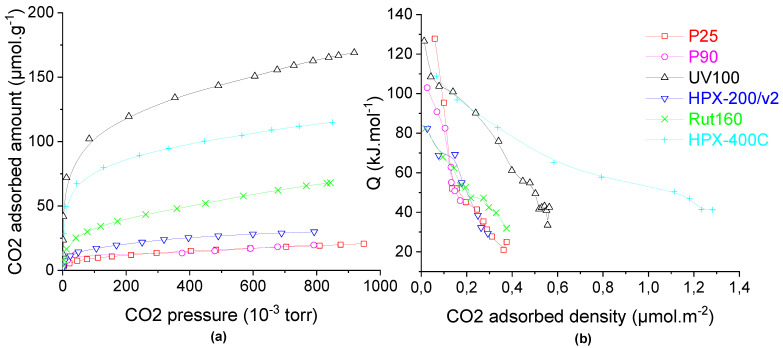
Carbon dioxide adsorption on TiO_2_ (**a**) isotherms and (**b**) calorimetric curves. Conditions: TiO_2_ samples pre-treated at 150 °C under secondary vacuum for 5 h, CO_2_ adsorption at 30 °C.

The catalysts’ basicity was studied in the gas phase by carbon dioxide adsorption monitored by microcalorimetry. The CO_2_ adsorption isotherms (Figure 5a) show that anatase UV100, which has the highest BET surface area (300 m^2^.g^−1^), has the highest amount of basic sites (120 µmol.g^−1^), then the rutile catalysts (HPX-400C and Rut160) have an intermediate basicity (85 and 40 µmol.g^−1^, respectively) not correlated with their surface areas (85 vs. 175 m^2^.g^−1^). Anatase HPX-200/v2 and anatase/rutile catalysts (P25 and P90) had the lowest basic sites amounts, <25 µmol.g^−1^. Since the different TiO_2_ samples have different surface areas, the calorimetric curves are also displayed as a function of the basic sites’ densities (basic sites amount divided by the catalyst’s surface area). HPX-400C (rutile) and UV100 (anatase) showed by far the highest basic sites density with a significant proportion of very weak basic sites in the case of HPX-400C, with Q_diffCO2_ between 40 and 80 kJ.mol^−1^. As shown in Table 2, the other catalysts had lower basic sites densities, <0.23 µmol.m^−2^.

The calorimetric curves of the six catalysts (Figure 5b) showed a progressive decrease of CO_2_ adsorption’s heat with the CO_2_ coverage which indicates the presence of basic sites with different strength, i.e., quite heterogeneous surfaces in terms of basic strength.

From these values, one can conclude that there is no correlation between either the basic properties and the TiO_2_ structure or the crystallites sizes and specific surface areas.

However, the superficial acid/base balance, reported in Table 2, seems to differentiate the anatase from the rutile phase: the rutile TiO_2_ surfaces appear to exhibit the lowest acid/base balance with a ratio close to 5; while the highest acid/base ratio, between 7.5 and 27, is measured for the pure anatase or the anatase/rutile mixture. In agreement with these remarks, P25, which contains 20% of the rutile phase, presents a lower acid/base balance than P90, which contains only 8% of the rutile phase.

Note that the surface acid/base balance does not seem to depend on the morphology of the TiO_2_ samples nor to the presence of impurities such as S or Cl traces (Table S1).

### 2.4. DHA Conversion in Water

As discussed above, the model reaction of DHA conversion in water was investigated in the presence of the six TiO_2_ samples with different original acid base properties as characterized above in the gas phase. Our initial goal was to determine, a priori, their Lewis acid sites water tolerance since it is largely accepted that DHA conversion would proceed via an acid catalyzed cascade transformation producing first pyrulvaldehyde and then lactic acid in the presence of water-tolerant Lewis acid catalysts. Scheme 1 represents the largely accepted mechanism of DHA transformation.

**Figure 6:**
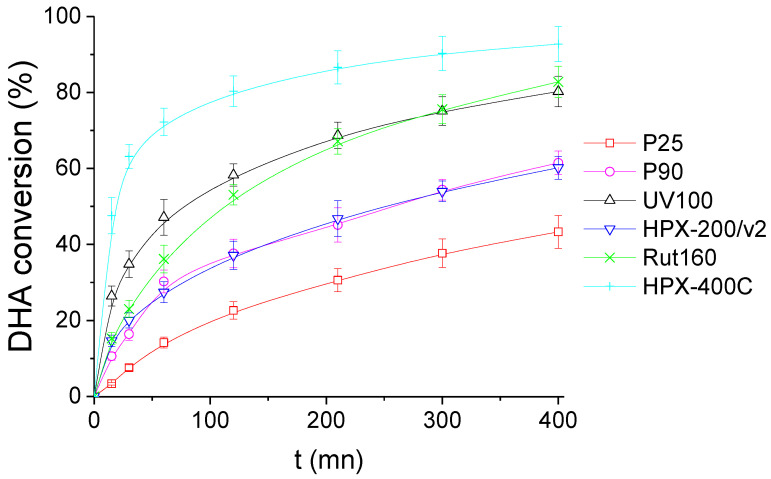
DHA conversion with the time course of the reaction in the presence of the six TiO_2_. Conditions: T = 90 °C, P_air_ = 1 atm, V_water_ = 200 mL, (DHA) = 0.1 mol.L^−^^1^, (TiO_2_) = 10 g.L^−^^1^.

Figure 6 shows the DHA conversion as a function of time in the presence of the six TiO_2_ catalysts. Among the most active TiO_2_ samples, pure rutile HPX-400C and Rut160 led to DHA conversions around 90% and 80%, respectively, after 400 min of reaction, and UV100 (pure anatase) converted also ~80% of DHA during the same reaction time. The anatase/rutile mixture, P25, appears as the less active TiO_2_, leading to the DHA conversion of ~40% at the reaction end. The other anatase/rutile mixture (P90) is slightly more active, with ~60% DHA conversion after 400 min, similar to HPX-200/v2 (pure anatase TiO_2_).

From the TiO_2_ activity ranking demonstrated in Figure 6 and the acid-base properties summarized in Table 2, the correlation with the basicity is highlighted: the TiO_2_ samples with the lowest acid/basic sites balance, i.e., HPX-400C, Rut160 and UV100, are the most active. Moreover, one can conclude for the absence of any correlation with acidity. Indeed, we searched possible correlations between the initial rate of DHA conversion and the total amount of acid sites (µmol.g^−1^) or the total acid sites densities (µmol.m^−2^) in order to take into account the variability of the samples’ surfaces, but no link was observed between the initial rate of DHA conversion and the original acidity of the samples determined in the gas phase (Figures S1 and S2).

We think that these results are of peculiar significance. Indeed, the absence of a correlation between the DHA conversion and the original acidity of the TiO_2_ samples determined in the gas phase might be explained by the variability of the acid sites water tolerance as a function of the TiO_2_ crystallinity or morphologies.

**Figure 7:**
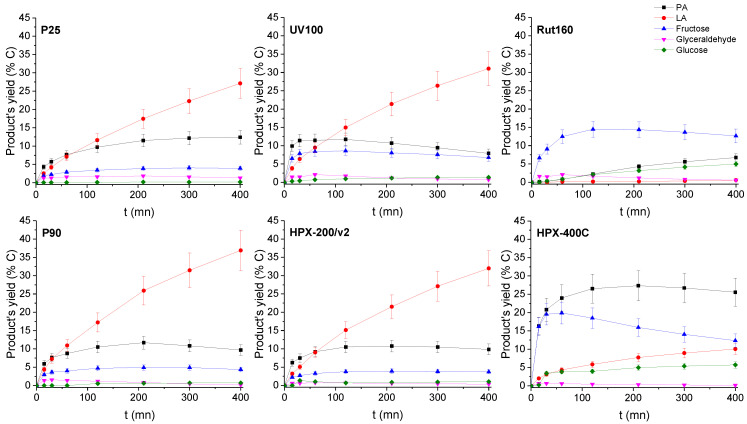
Products’ yield time course in the presence of the six TiO_2_s. Conditions: T = 90 °C, P_air_ = 1 atm, V_water_ = 200 mL, (DHA) = 0.1 mol.L^−1^, (TiO_2_) =10 g.L^−1^.

The evolutions of the products’ yields with time or with the DHA conversion are presented in Figure 7 and Figure S3, respectively. 

**Figure 8:**
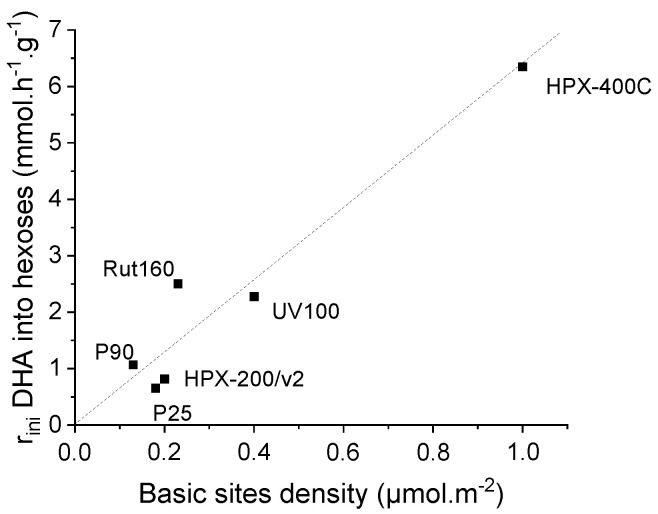
Increase of initial rate of hexoses formation as a function of TiO_2_′s basic sites density.

In the presence of the most active catalysts, HPX-400C, Rut160 and UV100, the formation of fructose appears as a main initial pathway in the transformation of DHA in addition to PA formation. However, whereas fructose is the main primary product in the presence of Rut160, fructose and PA are two primary products formed in equivalent amount in the presence of HPX-400C, and the formation of PA is initially slightly favoured with respect to fructose in the presence of UV100. In the presence of all the other TiO_2_ (HPX-200/v2, P25 and P90), the initial formation of PA and its further conversion into LA is the main pathway. With the reaction progress over the first three catalysts, the fructose yield declines and we can observe a parallel increase of the glucose formation, explained by the base catalyzed fructose–glucose isomerization. The initial formation of fructose well agreed with the observed link between the TiO_2_ samples’ activity and the basicity of their surface. Indeed, fructose, which is C6 sugar, can be formed by aldolic condensation of the trioses DHA and glyceraldehyde, easily formed by DHA isomerization. Note that glyceraldehyde is detected as minor product. Both reactions, aldolic condensation and the aldo-ketose isomerization, responsible for fructose–glucose and DHA–glyceraldehyde isomerization, are base catalyzed steps [36,37]. Accordingly, a linear increase of initial rates of hexoses formation with the basic sites densities can be drawn (Figure 8). Apparently, the presence of micropores in Rut160 does not interfere with the trioses condensation reaction.

Besides, the less active materials (P25, P90 and HPX-200/v2) presented activities consistent with the previous art: the initial formation of PA from DHA dehydration, which slows down with the reaction progress while the LA yield continuously increased (Figure 7). This fits well with the generally accepted mechanism which involves, first, an acid catalyzed (Brønsted or Lewis) DHA dehydration, and then, its rehydration into LA in the presence of water-tolerant Lewis acids sites [16,19]. However, it is interesting to note that LA is rapidly formed. At a DHA conversion lower than 10%, the amount of LA is already practically equivalent to that of PA. Such a kinetic profile was already reported over a solely water-tolerant Lewis acid catalyst such as Sc(OTf)_3_ (OTf = triflate) [38]. Most likely, the two steps, DHA dehydration into PA and its further rehydration into LA, occur solely on the Lewis acid sites of TiO_2_ that have kept efficiency in water. Presumably, this would suggest that the successive transformation of PA into LA would not be the rate-limiting step when only Lewis acids sites are involved. LA achieved a maximum yield of ~40% over P90 after 6 h corresponding to a selectivity of about 60% at a DHA conversion of 60% against less than 5% for pure rutile. Note that fructose, glucose, and glyceraldehyde were detected over all TiO_2_ samples but in low proportions over the less active catalysts. The aldo-ketose isomerization was reported to be catalyzed as well by basic or Lewis acid sites [36,37,39].

**Figure 9:**
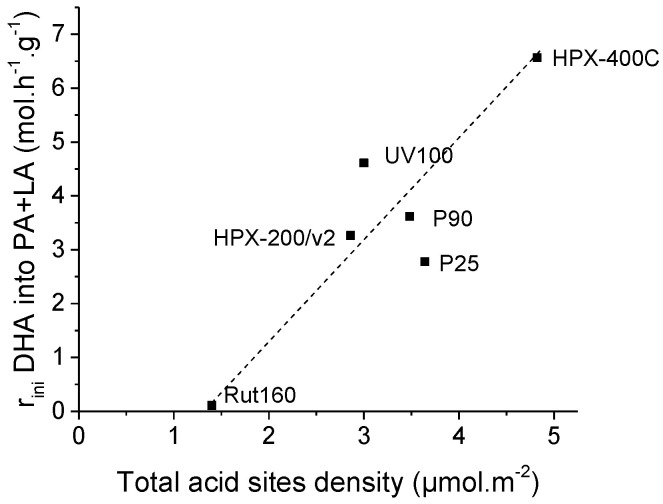
Increase of initial rates of pyruvaldehyde and lactic acid formation with TiO_2_′s total acid sites density.

Again, we tried to highlight an expected relationship between the initial rates of PA and LA formation and the catalyst acidity. Figure S4 evidences the absence of correlation between the initial rate of PA and LA formation with the total amount of acid sites determined in gas phase expressed per g of catalyst. The initial formation of PA and LA matches more with the total acid site density determined in gas phase and expressed in µmol per m^2^: Figure 9 shows a progressive increase of the initial rates of PA and LA formation with the rise of the total acid sites densities. 

This could have various explanations:

(1) A surface with enhanced proximity between the acid sites could be less inhibited by water adsorption if the DHA adsorption would require several adsorption sites. In other words, a surface with a higher density of Lewis acid sites would favor the competitive adsorption of triose sugars as regards to water, i.e., this type of surface would be more water tolerant when a triose sugar conversion is concerned. This observation is consistent with our previous observation of the improved adsorption of glucose on TiO_2_ samples, which have a high density of original Lewis acid sites (determined in the gas phase) [40].

(2) Disagreeing with most of the previous literature, this could also indicate that DHA dehydration–isomerization into pyruvaldehyde and lactic acid could proceed via a bimolecular mechanism with the intermediate formation of dimeric intermediates as suggested in a recent paper [23].

**Figure 10:**
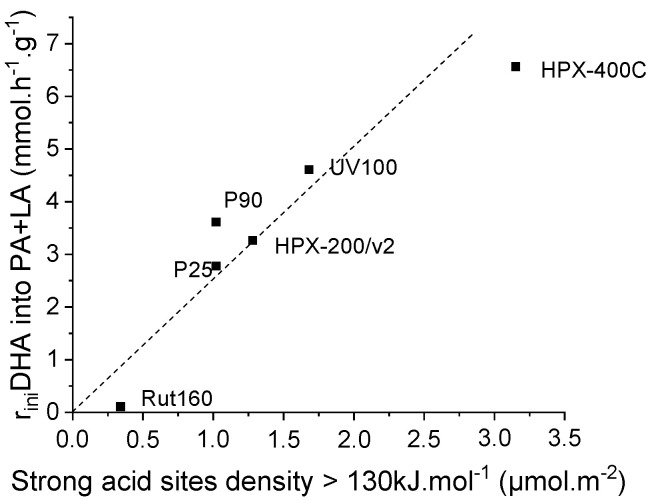
Linear increase of the initial rates of pyruvaldehyde and lactic acid formation with TiO_2_′s original strong acid sites density (sites with heat of ammonia adsorption > 130 kJ.mol^−^^1^ as determined by calorimetry of ammonia adsorption).

It is also worth noticing that the dashed linear line, which is simply a guide for eyes to highlight the progressive increase of initial PA and LA rates formation, does not pass through the origin point but crosses the *X*-axis at a value ~1.5 µmol.m^−2^. Presumably, this could be consistent with the assumption that the water tolerance of the TiO_2_ surface as regards to triose sugar adsorption, and then its conversion would require a minimum density of Lewis acid sites or acid strength. In fact, when we plot the initial rate of PA and LA formation against the acid sites’ density of the strongest original acid sites, characterized by a heat of ammonia adsorption higher than 130 kJ.mol^−1^ as determined by microcalorimetry, the dashed line goes through the origin (Figure 10).

This better correlation would suggest that an additional parameter would intervene for a more efficient adsorption/conversion of triose sugar in water. The higher the strength of the original Lewis acid sites, the more efficient would be the coordination of triose sugar as regards to neutral water. Below these limits, an acid sites density of ~1.5 µmol.m^−2^ and an acid strength of 130 kJ.mol^−1^, the water/triose competitive adsorption would be favorable to water adsorption and the TiO_2_ surface would appears as less water tolerant.

**Figure 11:**
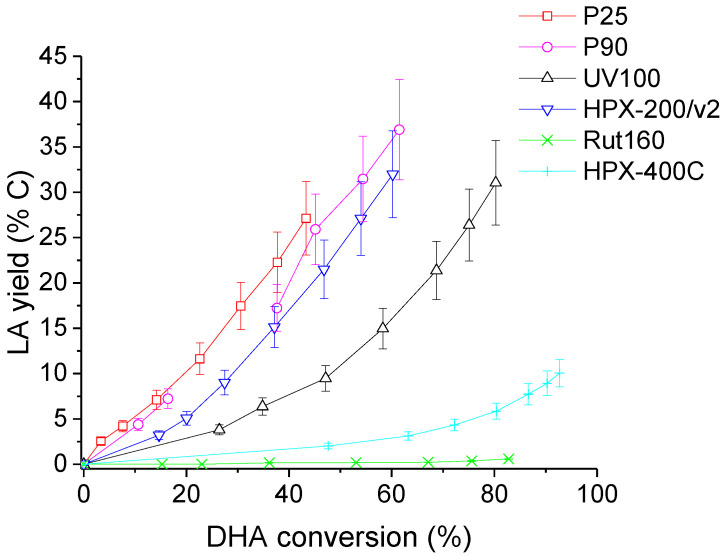
Evolution of lactic acid (LA) yield with DHA conversion in the presence of the six TiO_2_. Conditions: T = 90 °C, P_air_ = 1 atm, V_water_ = 200 mL, (DHA) = 0.1 mol.L^−^^1^, (TiO_2_) = 10 g.L^−^^1^.

At last, LA yields as a function of DHA conversion are illustrated in Figure 11. It may be seen that, at a given DHA conversion, LA yield was the highest for P25, intermediate for the other anatase TiO_2_ catalysts (pure or mixtures) and almost nil for rutile TiO_2_ (Rut160 and HPX-400C).

To confirm the observed acidity/basicity effect on DHA transformation in the presence of the TiO_2_ catalysts, a well-known water-tolerant solid acid (Lewis and Brønsted acid sites), NbOH [38,41] and a basic heterogeneous catalyst, MgLaO [42,43], were used to convert DHA.

**Figure 12:**
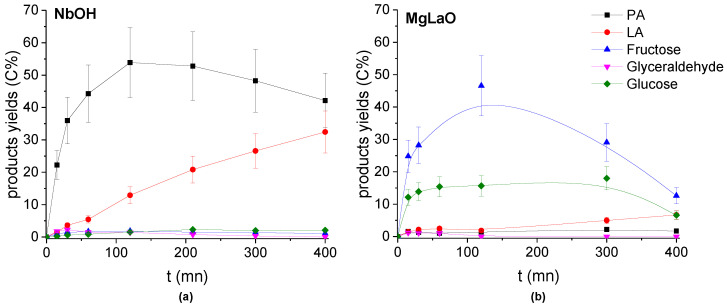
Evolution of the Products’ yields with the reaction time in the presence of (**a**) NbOH and (**b**) MgLaO. Conditions: T = 90 °C, P_air_ = 1 atm, V_water_ = 200 mL, (DHA) = 0.1 mol.L^−^^1^, (catalyst) = 10 g.L^−^^1^.

NbOH (Figure 12) exhibited an activity close to the most acid TiO_2_ sample (Figure 7) in water, where PA was initially formed from DHA dehydration and then its formation slowed down with the reaction progress while the LA yield increased continuously. NbOH produced more PA (maximum yield of 55 C%) (Figure 12) than the most acid TiO_2_ catalyst (maximum yield around 15 C%) (Figure 7).

On the contrary, MgLaO exhibited an activity similar to the most basic TiO_2_ (Figure 12) where the main products of DHA conversion were the condensation products: fructose and glucose. PA and LA were formed with very low yields.

**Figure 13:**
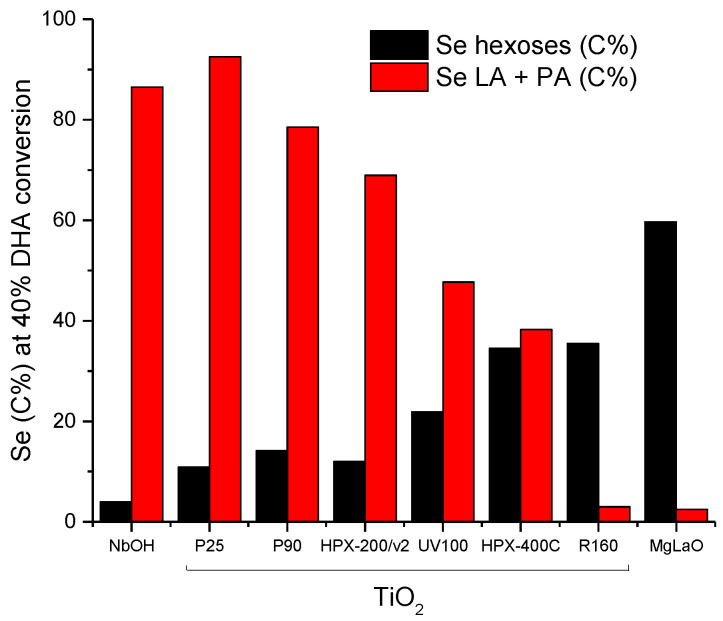
Evolutions of hexoses selectivity and Pyruvaldehyde/lactic acid (PA + LA) selectivities at 40% DHA conversion as a function of the TiO_2_′s acid/base balance. Comparison with the reference solid acid, NbOH, and the reference solid base, MgLaO.

Finally, we plotted hexoses selectivities and the sum of PA and LA selectivities at an equivalent DHA conversion level (40%) in Figure 13. The progressive increase of hexoses selectivities against the progressive decrease of PA + LA selectivities is observed. This opposite behavior appears to be roughly linked to the original acid/base balance measured on the TiO_2_ samples. The rutile TiO_2_, characterized by the lowest acid/base balance (Table 2), was the most selective for hexoses formation among the TiO_2_ samples with a behavior close to that of the reference basic catalyst MgLaO. On the other hand, P25 with the highest acid/base balance was the most selective for PA and LA formation close to the selectivity observed over NbOH, the reference acid catalyst.

So, these results show that DHA conversion can be converted into LA and PA by the catalysts’ acid sites and/or to condensation products (fructose, glucose) by the catalysts’ basic sites. This reaction appears as a suitable model reaction to characterize any catalysts’ acid and basic sites in water.

**Scheme 2:**
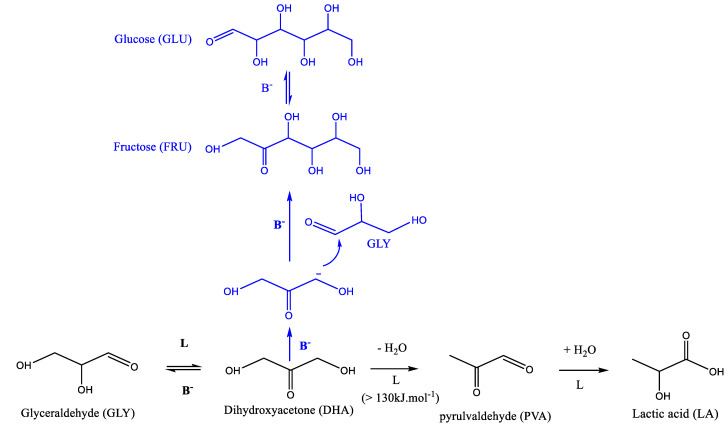
Proposed mechanism which would prevail on bifunctional acid-base catalysts.

To rationalize all the above reported correlations between the original acid or base properties of the TiO_2_ samples and their activities/selectivities for DHA conversion in water, a mechanism is proposed (Scheme 2). This mechanism involves two parallel pathways: one catalyzed by the catalysts’ basic sites leading to fructose and glucose and the other one promoted by the strongest Lewis acid sites leading to DHA isomerization into LA. The equilibrium between these two pathways depends on the superficial acid/base balance of the catalyst.

## 3. Materials and Methods

### 3.1. Commercial Titanium Dioxide (TiO_2_)

Six commercial TiO_2_ with different crystalline phases and different specific surface areas were tested: P25 and P90, which are mixture of anatase and rutile, provided by Evonik, two pure anatase: UV100 from Hombikat and HPX-200/v2 from Cristal and two pure rutile: Rut160 from Nanostructured and Amorphous Materials Incorporation and HPX-400C from Cristal. Rut160 was calcined in a muffle oven at 400 °C during 20 h to eliminate organic pollution from its surface. All the other catalysts were used without any treatment. The main impurities of the TiO_2_ samples were summarized in the Table S1.

### 3.2. X-ray Diffraction (XRD)

The catalysts’ crystalline structures and the sizes of their crystallites were analyzed by XRD using a Bruker D8 Advance diffractometer A25 equipped with a copper anode. Powdered samples were analyzed within a 2θ range of 4–80° at 0.02° per step and with an acquisition time of 96 s per step. The IRCELYON’s XRD Service collected the diffractograms.

### 3.3. Brunauer–Emmett–Teller Surface Area (SBET) Analysis and Pores’ Size Distribution

The catalysts’ texture was characterized using nitrogen adsorption/desorption isotherm at −196 °C in a Micromeritics ASAP 2020 device. Before the analysis, the catalysts were degassed at 300 °C (heating rate 5 °C.min^−1^) during 2 h.

### 3.4. Pyridine Adsorption Monitored by Fourier Transform Infrared Spectroscopy (FTIR)

The acid site types present on the catalysts (Lewis and/or Brønsted) were identified by the adsorption of pyridine monitored by FTIR. FTIR spectra were recorded with a Brucker Vector 22 spectrometer in absorption mode with a resolution of 2 cm^−1^. Catalysts powders were pressed into self-supported pellets. The pellets were placed in an IR pyrex cell equipped with CaF_2_ windows and connected to a first vacuum line allowing thermal pretreatment in the absence of pyridine pollution, then the cell was connected to a second piece of vacuum pyrex equipment to perform pyridine adsorption and desorption. All the samples were treated under flowing synthetic air (2 L.h^−1^) for one hour at 150 °C, then under the secondary vacuum while cooling for one hour. Then, pyridine was adsorbed under saturation vapor pressure at ambient temperature. The pyridine was desorbed for one hour, firstly at ambient temperature, then at 150 °C for 1 h (to remove the physisorbed pyridine species on the catalyst). FTIR of pyridine adsorption was only used to discriminate between Lewis and Brønsted acid sites: the 19b vibration mode allows one to discriminate pyridine coordinated to Lewis acid sites (1450 cm^−1^) from the pyridinium ion (1550 cm^−1^). In the presence of physisorbed pyridine molecule, this vibration mode is observed at 1438 cm^−1^ [35].

### 3.5. NH_3_ Adsorption Monitored by Microcalorimetry

The number and strength of acid sites of the catalysts were measured by ammonia adsorption at 80 °C, monitored by microcalorimetry using a Tian–Calvet calorimeter (MS80 Setaram) coupled with a volumetric equipment for isotherms measurement. This technique allows one to measure the differential heats of adsorption evolved for small and known amounts of ammonia molecules adsorbed on the catalyst surface. This energy depends on the strength of acid sites present on the catalyst surface. The total amount of acid sites is deduced from the isotherms (intersection of the tangent to the *Y*-axis). Thirty mg of each catalyst were placed in a glass cell and pretreated at 150 °C for 5 h under a secondary vacuum (heating rate of 2 °C.min^−1^). The cell was then placed in the Tian–Calvet calorimeter stabilized at 80 °C for a night and coupled with a volumetric equipment. Finally, successive doses of ammonia were brought into contact with the catalyst while the heats of adsorption were recorded.

### 3.6. CO_2_ Adsorption Monitored by Microcalorimetry

The number and strength of the basic sites of the catalysts were measured by the same calorimetric technique but using carbon dioxide as a probe, and the adsorption was done at 30 °C. The catalysts (50 mg) were treated exactly as for the acid sites quantification, following the same pretreatment procedure.

The total amount of basic sites is deduced from the isotherms (intersection of the tangent to the *Y*-axis).

### 3.7. Dihydroxyacetone (DHA) Conversion in Water

The reactions were conducted in a three-neck round bottom flask (250 mL) equipped with a water condenser, a temperature sensor and a rubber septum. At first, water and catalyst (10 g.L^−1^) were placed in the flask (total volume of 200 mL) and heated at 90 °C in a controlled temperature oil bath and agitated by a magnetic stirrer. When the temperature stabilizes, DHA powder (1,3-dihydroxyacetone dimer, >97%, purchased from Sigma-Aldrich) was added (t = 0) and the reactions were conducted during 400 min. The DHA initial concentration was 0.1 mol.L^−1^. Samples were taken with a 1 mL syringe through the septum and analyzed by high-performance liquid chromatography (HPLC): a Shimadzu Prominence system equipped with a refractive index detector (RID) and a COREGEL 107H column maintained at 40 °C to quantify the products and the residual substrate (DHA) (Figure S5). The mobile phase was acidified water (H_2_SO_4_ 1.7 mM) with a flow of 0.6 mL.min^−1^.

Two other non TiO_2_ catalysts were tested as references for this reaction: a commercial Nobium hydroxide (NbOH) from CBMM, Brazil, and a home-made MgLaO [42].

## 4. Conclusions

Contrary to all expectations, the most active TiO_2_ catalyst for DHA conversion has the most basic surface, as established by calorimetry in the gas phase. The formations of hexoses from DHA on these samples indicates the occurrence of a parallel pathway catalyzed by basic sites. In agreement with the previous literature, lactic acid formation is thought to be linked to the sample’s original Lewis acidity.

This was observed on various TiO_2_ samples with a variable superficial acid/base balance, and that was confirmed over two reference catalysts: an acid one NbOH and a basic one MgAlO.

Moreover, based on the original acid-base properties of the TiO_2_ samples determined in the gas phase and the results obtained studying this model reaction in water, our results suggest that:

- The water tolerance of TiO_2_ superficial acidity as regards to triose conversion would require a minimum Lewis acid sites density with enough acid strength (Q_diffNH3_ > 130 kJ.mol^−1^).
- The TiO_2_ basic sites would be water tolerant whatever their original basic strength.

## Figures and Tables

**Table 1:** Textural features and particle sizes of commercial TiO_2_ samples.

Catalysts	S_BET_^1^ (m^2^.g^−1^)	Mesopores Size ^2^	S Micropore ^3^ (m^2^.g^−1^)	Crystallites Size ^4^ (nm)
P25 (80% anatase/20% rutile)	55	>4 nm	-	18.1
P90 (92% anatase/8% rutile)	115	>5 nm	-	11.6
UV100	300	>3 nm	194	7.5
HPX-200/v2	96	>4 nm	-	13.1
Rut160	175	>4 nm	82	8.5
HPX-400C	85	>5 nm	-	16.4

^1^ Pretreatment: 2 h at 300 °C under vacuum (heating rate: 5 °C.mn^−1^), ^2^ BJH, ^3^ t-plot, ^4^ from XRD patterns.

**Table 2:** Catalysts’ acid/basic sites amounts and densities.

Catalyst	Acid Sites Amount (µmol.g^−1^)	Acid Sites Density (µmol.m^−2^)	Basic Sites Amount (µmol.g^−1^)	Basic Sites Density (µmol.m^−2^)	Acid/Base Sites Ratio
P25	200	3.6	10	0.18	20
P90	400	3.5	15	0.13	27
UV100	900	3	120	0.4	7.5
HPX-200/v2	275	2.9	20	0.2	13.7
Rut160	240	1.4	40	0.23	6
HPX-400C	410	4.8	85	1	4.8

Conditions: TiO_2_ samples pretreated at 150 °C under secondary vacuum for 5 h, NH_3_ adsorption performed at 80 °C, CO_2_ adsorption performed at 30 °C.

## Data Availability

The data presented in this study are available on request from the corresponding author.

## Supplementary Materials

The following supporting information can be downloaded at: https://www.mdpi.com/article/10.3390/molecules27238172/s1, Table S1: TiO_2_ samples’ impurities quantified by X Fluorescence analysis (wt%); Figure S1: Initial rate of DHA conversion as a function of TiO_2_’s acid sites amount determined in the gas phase; Figure S2: Initial rate of DHA conversion as a function of TiO_2_’s acid sites density determined in the gas phase; Figure S3: Evolution of products’ yields with DHA conversion in the presence of the six TiO_2_s, NbOH and MgLaO; Figure S4: Initial rate of pyruvaldehyde (PA) and lactic acid (LA) formation as a function of TiO_2_’s acid sites amount determined in the gas phase; Figure S5: HPLC Chromatograph of products after reaction for 400 min catalyzed by P25 (1 glucose, 2: fructose, 3: glyceraldehyde, 4: pyruvaldehyde, 5: lactic acid, 6: dihydroxyacetone).

## Sample Availability

Samples are available on request from the corresponding author.
