# Supplementary material for: TiO2 Catalyzed Dihydroxyacetone (DHA) Conversion in Water: Evidence That This Model Reaction Probes Basicity in Addition to Acidity
Source: Molecules. 2022 Nov 24;27(23):8172. doi: 10.3390/molecules27238172 (PMC9736615; doi:10.3390/molecules27238172)
Supplement: Supplementary file 1 [file molecules-27-08172-s001.zip › molecules-2031324-supplementary.pdf]

Supplementary Materials

TiO_2 catalyzed dihydroxyacetone (DHA) conversion in water: evidences that this model reaction probes basicity in addition to acidity

Insaf Abdouli, Frederic Dappozze, Marion Eternot, Chantal Guillard, Nadine Essayem*

University Claude Bernard, CNRS, IRCELYON, UMR5256, Villeurbanne 69626 (France)

* nadine.essayem@ircelyon.univ-lyon1.fr

Table S1: TiO_2 samples' impurities quantified by X Fluorescence analysis (wt%)

impurities	P	Cl	Si	S	C
P25	0.09	**0.17**	-	-	-
P90	0.09	**0.20**	-	-	-
UV100	0.22	0.01	0.02	**0.11**	-
HPX-200/v2	0.10	0.07	0.01	-	-
Ru160	0.26	-	**5.00**	**0.32**	**1.61**
HPX-400C	0.10	0.07	0.01	-	-

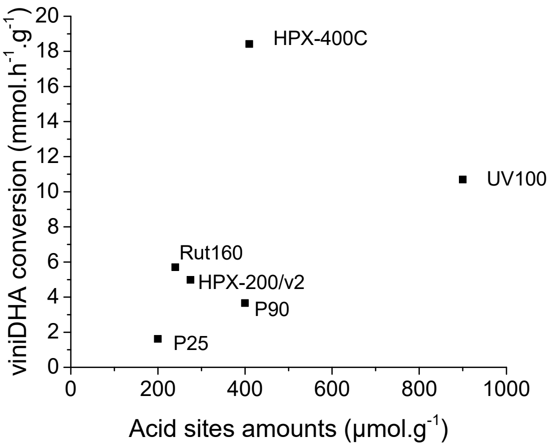

Figure S1. Initial rate of DHA conversion as a function of TiO_2's acid sites amount determined in gas phase.

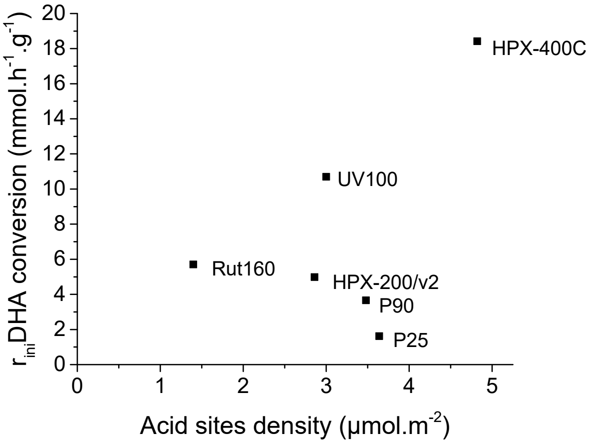

Figure S2. Initial rate of DHA conversion as a function of TiO_2's acid sites density determined in gas phase.

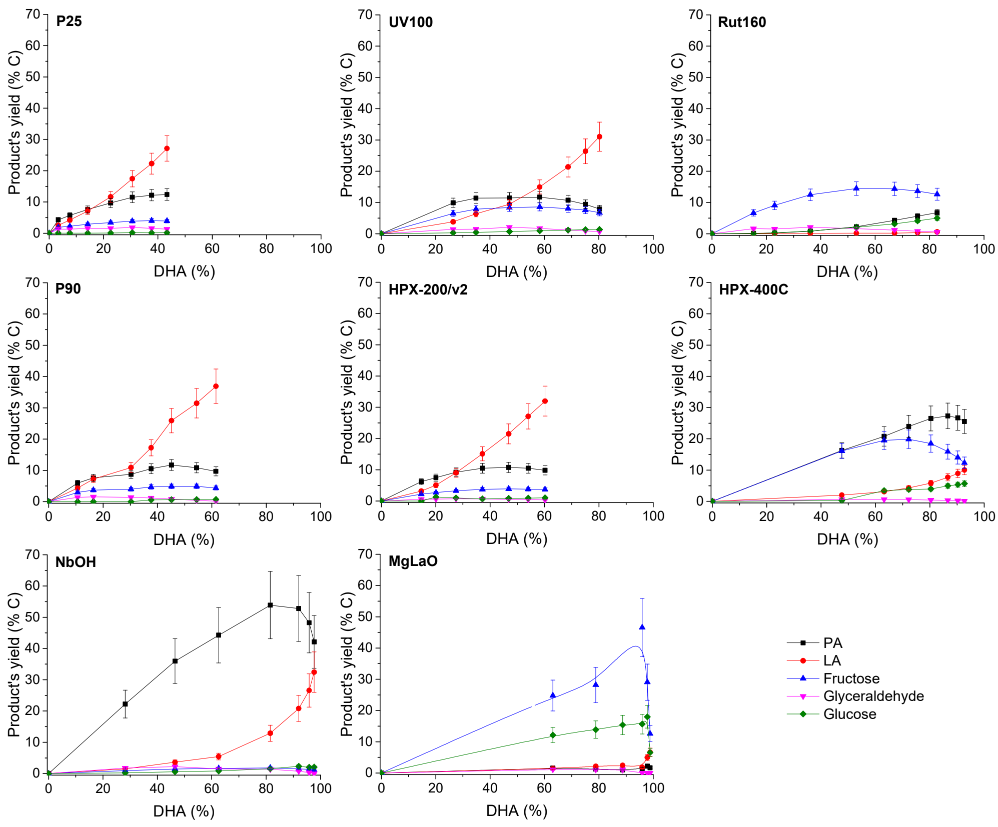

Figure S3. Evolution of products' yields with DHA conversion in the presence of the six TiO_2s, NbOH and MgLaO. Conditions: T= 90 °C, Pair=1 atm, Vwater= 200 mL, [DHA] = 0.1 mol.L^{-1}, [catalyst] =10 g.L^{-1}.

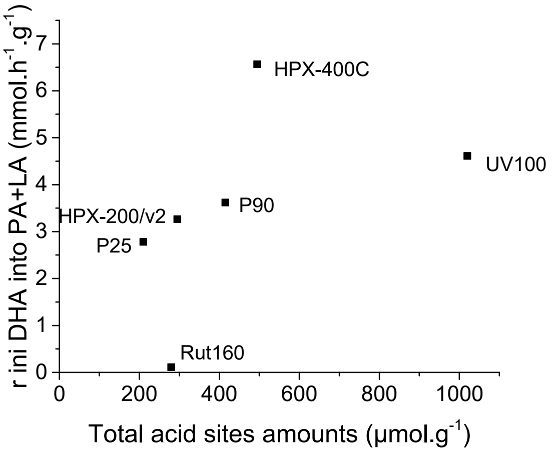

Figure S4. Initial rate of pyruvaldehyde (PA) and lactic acid (LA) formation as a function of TiO_2's acid sites amount determined in gas phase.

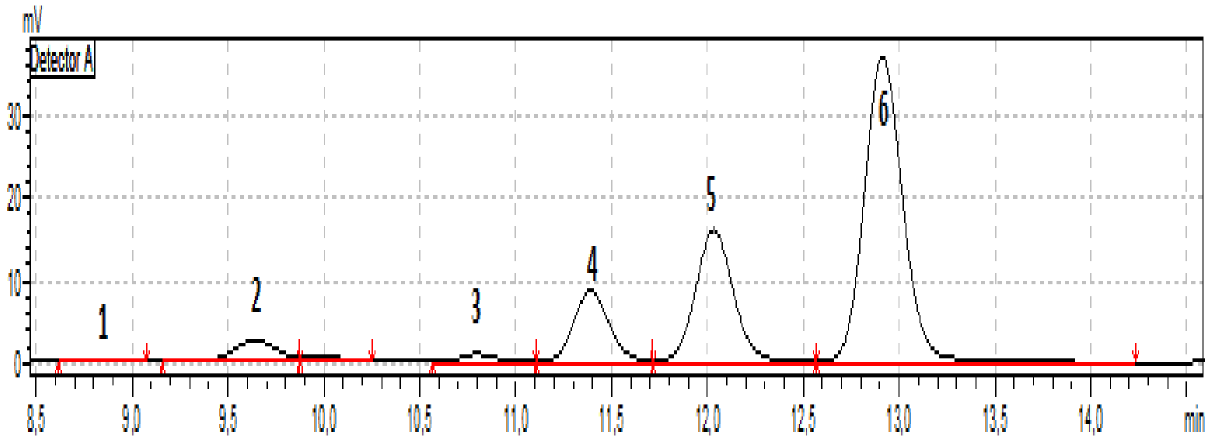

Figure S5. HPLC Chromatograph of products after reaction for 400 minutes catalyzed by P25 (1: glucose, 2: fructose, 3: glyceraldehyde, 4: pyruvaldehyde, 5: lactic acid, 6: dihydroxyacetone).
